# Pennsylvania Hospital—Surgical Wards—Service of Dr. Norris

**Published:** 1850-12

**Authors:** 


*Pennsylvania Hospital—Surgical Wards—Service of* Dr. Norris.

*Cases Discharged from Feb. 1st to March 1st,* 1850.

| Disease. | Cured. | Average time under treatment in days | By request. | Average time under treatment in days | Died. | Average time under treatment in days | Tot. |
|---|---|---|---|---|---|---|---|
| Abscess - - | 1 | 72 | 0 | 0 | 0 | 0 | 1 |
| Burns - - | 1 | 33 | 0 | 0 | 3 | 4.7 | 4 |
| Calculus - - | 1* | 81 | 0 | 0 | 0 | 0 | 1 |
| Contusion - | 1 | 24 | 0 | 0 | 0 | 0 | 1 |
| Erysipelas - | 1 | 24 | 0 | 0 | 0 | 0 | 1 |
| Fractures, 13 | | | | | | | |
| simple 10, viz.: | | | | | | | |
| Humerus - | 1 | 34 | 0 | 0 | 0 | 0 | 1 |
| Condyle of humerus | 1 | 28 | 0 | 0 | 0 | 0 | 1 |
| Radius - - | 4 | 43.5 | 0 | 0 | 0 | 0 | 4 |
| Radius and ulna | 1 | 34 | 0 | 0 | 0 | 0 | 1 |
| Femur - - | 2 | 71 | 0 | 0 | 0 | 0 | 2 |
| Leg - - | 1 | 86 | 0 | 0 | 0 | 0 | 1 |
| Compound 3, viz.: | | | | | | | |
| Skull - - | 1 | 22 | 0 | 0 | 0 | 0 | 1 |
| Leg - - | 2 | 104.5 | 0 | 0 | 0 | 0 | 2 |
| Gangrene - | 1 | 130 | 0 | 0 | 0 | 0 | 1 |
| Gonorrhœa - | 2 | 42 | 1 | 13 | 0 | 0 | 3 |
| Hæmorrhois - | 1 | 35 | 0 | 0 | 0 | 0 | 1 |
| Hernia, strangulated | 0 | 0 | 0 | 0 | 1 | 3 | 1 |
| Hydrocele - | 0 | 0 | 1 | 3 | 0 | 0 | 1 |
| Inflammation of face | 0 | 0 | 1 | 13 | 0 | 0 | 1 |
| " hip | 0 | 0 | 1 | 80 | 0 | 0 | 1 |
| " leg | 1 | 22 | 0 | 0 | 0 | 0 | 1 |
| Luxation - - | 1† | 3 | 0 | 0 | 0 | 0 | 1 |
| Sub-luxation - | 2 | 18.5 | 0 | 0 | 0 | 0 | 2 |
| Paronychia - | 1 | 10 | 0 | 0 | 0 | 0 | 1 |
| Syphilis - - | 1 | 25 | 1 | 65 | 0 | 0 | 2 |
| Tumor - - | 2‡ | 21.5 | 0 | 0 | 0 | 0 | 2 |
| Wounds, 7, viz.: | | | | | | | |
| Gunshot - - | 1 | 89 | 0 | 0 | 1 | 0 | 1 |
| Incised - - | 1 | 29 | 0 | 0 | 0 | 0 | 1 |
| Lacerated - | 3 | 16.7 | 0 | 0 | 0 | 0 | 3 |
| Punctured - | 2§ | 25.5 | 0 | 0 | 0 | 0 | 2 |
| | 37 | | 5 | | 4 | | 46 |

*By lithotomy.
†Of humerus.
‡By extirpation.
§One of the thigh near to the femoral artery and complicated with delirium tremens.

